# Clinical Applications of Heart Rate Variability in the Triage and Assessment of Traumatically Injured Patients

**DOI:** 10.1155/2011/416590

**Published:** 2011-02-10

**Authors:** Mark L. Ryan, Chad M. Thorson, Christian A. Otero, Thai Vu, Kenneth G. Proctor

**Affiliations:** Divisions of Trauma and Surgical Critical Care, Ryder Trauma Center, Dewitt-Daughtry Family Department of Surgery, University of Miami Miller School of Medicine, 1800 NW 10th Avenue, Miami, FL 33136, USA

## Abstract

Heart rate variability (HRV) is a method of physiologic assessment which uses fluctuations in the RR intervals to evaluate modulation of the heart rate by the autonomic nervous system (ANS). Decreased variability has been studied as a marker of increased pathology and a predictor of morbidity and mortality in multiple medical disciplines. HRV is potentially useful in trauma as a tool for prehospital triage, initial patient assessment, and continuous monitoring of critically injured patients. However, several technical limitations and a lack of standardized values have inhibited its clinical implementation in trauma. The purpose of this paper is to describe the three analytical methods (time domain, frequency domain, and entropy) and specific clinical populations that have been evaluated in trauma patients and to identify key issues regarding HRV that must be explored if it is to be widely adopted for the assessment of trauma patients.

## 1. Introduction

Heart rate variability (HRV) is defined by the fluctuating time between normal sinus beats (RR intervals) [1] and indicates modulation of the heart rate by the autonomic nervous system (ANS) [2]. Afferent inputs from sensory and baroreceptors within the heart and great vessels, respiratory changes, vasomotor regulation, the thermoregulatory system, and alterations in endocrine function determine ANS influence on the heart [1].

In 1997, a consensus panel issued a set of guidelines regarding the measurement and interpretation of HRV [3]. Changes in HRV are now an accepted method of assessing autonomic dysfunction in patients in several pathologic states, with and without structural heart disease. In the 14 years since that report, there have been major technological advances and hundreds of publications in various patient populations, but there has been no comprehensive review specifically directed at trauma. This paper attempts to fill that gap.

It is now well established that absence of HRV is an early predictor of brain death [4, 5] and that low HRV correlates with increased mortality and morbidity after trauma [6–13]. Abnormal HRV is associated with increased intracranial pressure and decreased cerebral perfusion pressure [5, 9, 10, 14, 15]. Recently, it was suggested that HRV is a “new vital sign” and could be used as a trauma triage tool [7, 8, 11, 16, 17]. However, the mechanisms responsible for these associations are not clearly established, and no specific therapy is currently available to treat patients with abnormal HRV. Furthermore, there is no consensus on exactly how to measure HRV. Typically, it is quantified using at least one of three analysis domains: time, frequency, or entropy. Despite its enormous potential for assessment and triage, HRV has not been widely adopted in trauma patients. This paper will attempt to address the reasons for this and explore the major advances in various analytical techniques since the initial consensus report on HRV was issued in 1997.

## 2. Methods

### 2.1. Literature Search and Retrieval

The U.S. National Library of Medicine (Pubmed) Database was queried from January 1997 to August 2010 using the following keywords alone or in combination: “heart rate variability,” “trauma,” “volatility,” “complexity,” “entropy,” “heart period variability,” “autonomic,” “physiology,” “high frequency,” “low frequency,” “time domain,” “frequency domain,” “nonlinear dynamics,” and “triage.” Results of the electronic searches were supplemented by recommendation of peers and by reading reference lists of included studies.

### 2.2. Inclusion/Exclusion Criteria

Cohort studies, case control studies, and case series in the English language in adult or pediatric trauma patients were included in this paper. Studies not performed in human trauma patients or case reports were excluded.

## 3. Time Domain

### 3.1. Overview

This method subjects the integer heart rate or the R-R interval to basic statistical analysis (Table 1). Calculations which utilize interbeat (RR) intervals are taken from data sampled at a high rate (>100 Hz). Those derived from the integer HR are sampled less frequently (0.25–1 Hz). All time-domain variables provide information regarding global autonomic function but differ in whether they provide information on short-term variability (SDNN, RMSSD, and pNN50) or long-term variability (SDANN, HRVi) [3, 18].

### 3.2. Clinical Applications in Trauma

#### 3.2.1. Prehospital

In 2009, King et al. evaluated ECG data from 75 patients transported by helicopter to a level 1 trauma center to test if HRV could prospectively identify patients who would most benefit from urgent intervention. They reported that SDNN was a more accurate predictor of the presence of major injury and the need for life-saving operation than heart rate, systolic blood pressure (SBP), Glasgow Coma Scale (GCS), or paramedic judgment [16], but real-time data were not available.

#### 3.2.2. ICU

In 2004, Grogan et al. published a report on the Signal Interpretation and Monitoring (SIMON) project, a system implemented at Vanderbilt in 1998 for the continuous capture of physiologic data in patients admitted to the trauma ICU. They noted that heart rate volatility (which would later be referred to as integer HRV) and cardiac volatility-related dysfunction (later to be called uncoupling) during the first 24 hours of hospitalization were accurate independent predictors of morbidity and mortality, outperforming traditional vital signs [19]. Since its inception, published reports using data from this system have shown that HRVi and coupling predict mortality with as little as 12 hours of data [11, 20]. Increased uncoupling has been correlated with diminished physiologic reserve (defined as acidosis, coagulopathy, and hemorrhage severity), infection, multiple-organ system failure (MOSF), adrenal insufficiency, traumatic brain injury (TBI), and mortality [6, 11, 12, 20, 22]. These patients are particularly of interest, since they are at risk for multiple conditions associated with autonomic dysfunction such as sepsis, MOSF, adrenal insufficiency, as well as the frequent need for sedation and mechanical ventilation. A 2007 study by Proctor et al. showed that in patients assessed in either the ICU or the resuscitation bay, SDNN and RMSSD were correlated with the presence of TBI on computed tomography (CT) scan of the head as well as mortality [23].

### 3.3. Limitations

 There are several drawbacks specific to time-domain analysis. Although data acquired using the SIMON system is available continuously, HRVi and uncoupling are only capable of predicting outcome after data has been acquired for 12 or 24 hours, respectively. SDNN and RMSSD are both depressed by increases in heart rate [18], which suggests the possibility that decreases in these variables with morbidity and mortality are due to tachycardia in severely injured patients. However, these associations have not been shown when the mean heart rate is examined in these studies, suggesting that decreased time-domain variability is not solely a response to tachycardia. Time-domain analysis is also unable to distinguish between distinct biological signals [18], and more sophisticated measures such as frequency analysis are able to distinguish the effects of different ANS components on regulation of the heart rate.

## 4. Frequency Domain

### 4.1. Overview

This method involves analysis of the oscillations of RR intervals over time. Data are recorded at high frequency (>125 Hz) and the recording audited for ectopic beats and electrical artifact prior to analysis [24]. The digitized ECG tracing is analyzed such that the entire wave form is represented as the sum of periodic sine waves of different frequencies (Table 2) adjusted with respect to amplitude and phase so that the final sum replicates the original data [24]. This is done using mathematical processes such as continuous wavelet transformation (CWT), fast Fourier transform (FFT), and complex demodulation (CDM). The various energies that contribute to the heart rate impulse are grouped into three different peak levels, based upon their location on the power spectrum. Each peak corresponds with a different component of the ANS.

### 4.2. Clinical Applications in Trauma

#### 4.2.1. Prehospital

In 2006, Cooke et al. demonstrated that an increase in the HF/LF ratio was associated with increased mortality in a series of 42 patients transported by helicopter to a trauma center [7]. In a similar study evaluating the prehospital ECG data of 31 patients in 2009, Batchinsky et al. showed that HF amplitude (HFA) distinguished survivors from nonsurvivors with a data set as small as 100 beats [25]. HFA, an assessment of the amplitude of the oscillations in the HF range derived via CDM, was found to be a reliable predictor despite reductions in data set length or changes in patient status during the recording. This makes it a practical solution for the prehospital trauma setting where the average length of recording may be as little as one minute or 100 beats in length and the patient's condition is dynamic [25].

#### 4.2.2. TBI

The majority of the research on HRV in the frequency domain in trauma has focused on TBI patients. Goldstein et al. published several reports in children demonstrating suppression of LF and HF in association with brain death, decreased GCS, severity of neurologic injury, and poor outcome [4, 26]. Other studies in children have demonstrated that decreases in LF/HF are correlated with increases in intracranial pressure (ICP) greater than 30 mmHg, decreases in cerebral perfusion pressures (CPP) below 40 mmHg, and increased mortality [5]. Multiple subsequent studies in adults with TBI have shown that decreases in LF, HF, LF/HF, and total power (TP) are associated with brain death, increased mortality, decreased CPP, increased ICP, and poor outcome [10, 27–29]. Patient with decreased frequency-domain values in the postinjury period exhibited a prolonged duration of rehabilitation and neurologic recovery [30]. Regardless of the age group studied, frequency-domain variables have been proven to be indicative of severity and accurate predictors of outcome in TBI patients.

### 4.3. Limitations

Frequency-domain analysis is more sensitive to artifact or ectopy than the statistical time-domain methods. Since it is not feasible to screen patients for ectopy prior to monitoring in a trauma setting and ectopy has been shown to be common in healthy volunteers and trauma patients [32], this would appear to limit the utility of this method in the prehospital triage of trauma patients. Assumptions of stationarity and periodicity must be fulfilled, meaning that the overall condition of the patient must not change during the recording and the signal must be comprised of oscillations [18]. Long-term recordings of frequency-domain variables are not as useful, since the detailed information given about specific ANS components is obscured when recordings are averaged over long periods [3].

## 5. Entropy Domain

### 5.1. Overview

This method analyzes overall disorder or randomness in the ECG signal. It encompasses numerous methods which are summarized in Table 3. Nonlinear dynamic methods can be applied to the R-to-R interval to assess the complex variability present within the signal [33]. All measures of entropy are a global representation of autonomic nervous system functioning and complexity. The entropy methods quantify the probability of a repetitive pattern in the RR interval. If the next pattern can be predicted from the previous section, the signal is considered low in entropy and is therefore less complex [34]. The most commonly used variables are approximate entropy (ApEn) and sample entropy (SampEn), which are a reflection of the amount of irregularity in the R-to-R interval. Calculation of entropy can be accomplished with the use of proprietary software such as WinCPRS software (Absolute Aliens Oy, Turku, Finland) [13, 35, 36] and MATLAB 5.3 (the MathWorks, USA) [37] or by importing integer HR data into a publically available algorithm derived by Costa et al. at http://www.physionet.org/ [38, 39].

### 5.2. Clinical Applications in Trauma

#### 5.2.1. Prehospital

The first study with use of heart rate complexity in trauma in the prehospital setting originated at the Army Institute for Surgical Research [8]. A total of 117 patients were screened in the prehospital setting for ectopy-free, 800 beat ECG segments, but only 31 patients met criteria. The data showed that prehospital loss of RR interval complexity as measured by ApEn, SampEn, FDDA, and DFA distinguished survivors from nonsurvivors. ApEn outperformed traditional vital signs such as heart rate and blood pressure and was an independent predictor of in-hospital mortality [8]. In a follow-up study published two years later, the same cohort of patients was re-examined using slightly different methodology. They found that SampEn was consistently associated with mortality down to a data set size of 200 beats and was the only independent predictor of mortality [25]. These data suggested that SampEn may still be useful as an overall reflection of complexity in settings where longer sections of ECG cannot be obtained.

The same group conducted another study in the prehospital setting with regard to life saving interventions (LSI). LSI was defined as endotracheal intubation, cardiopulmonary resuscitation, cricothyroidotomy, and needle decompression. Complexity measures (ApEn, SampEn, FDDA, and DFA) were all lower in those patients who required LSI. SOD was higher in those patients, which is also consistent with decreased complexity. Because SampEn is relatively unaffected by a decrease in number of beats or RRIs, down to a data set of 200 beats, it may be more useful for emergency triage situations where only short segments of ECG data are available [43]. In 2010, Rickards et al. examined the use of HRV in identifying need for LSI in prehospital patients with normal initial vital signs. Out of multiple time-, frequency-, and entropy-domain variables evaluated only FDCL was associated with the need for LSI on multivariate analysis [17]. Before widespread application in the prehospital setting can be adopted, meaningful real-time interpretation of short interval data must be available.

#### 5.2.2. ICU

Intensivists have traditionally relied on sampling markers of hemodynamic and physiologic status at a single time point and can only compare these values with those collected at other discrete time points [44]. Because heart rate complexity data reflects overall balance of autonomic outflow, responsiveness, and neuroendocrine mechanisms, there is tremendous potential for the use in the care for critically ill patients [45]. Whereas other measures of HRV have correlated with multiple disease states including sepsis [46], multiorgan system dysfunction [29], and adrenal insufficiency [22], entropy has not been studied until recently.

Batchinsky et al. found that entropy, as measured by ApEn and SampEn, was lower in burn patients within 8 hours of admission to the intensive care unit [34]. Decreased ApEn has also been associated with mortality in acute TBI [37]. Norris et al. have investigated another measure of complexity in the intensive care unit, multiple-scale entropy (MSE). They found that MSE was significantly lower in nonsurvivors and was predictive of mortality using as little as 3 hours of heart rate data [36]. MSE measured within the first 24 hours was able to identify trauma patients at risk of in-hospital death [13]. Subsequent studies have correlated decreased MSE and beta-adrenergic receptor polymorphisms with increased mortality [35] and have shown that MSE predicts mortality independent of probability of survival based on location and mechanism of injury [45].

### 5.3. Limitations

Despite the overwhelming evidence of associations between heart rate complexity and numerous clinical outcomes, measurement and interpretation have limitations. A major constraint for clinical use is the fact that data needs to be analyzed off line with the use of proprietary software or algorithms. At this point, there is no way to conduct real-time evaluation of complexity data, and nearly all of the studies have been done retrospectively. In addition, analysis of ECG data requires that they be free of ectopy. If ectopic beats are encountered in a data set, the data must either be excluded, or the beat must be replaced via linear interpolation [33, 36]. The removal of patients with ectopic beats from analysis can introduce selection bias into the sample [3].

## 6. Summary

 There are at least 23 different variables using the 3 different methods of analysis that reflect HRV (Table 4), each with strengths and weaknesses. Decreases in HRV in trauma patients indicate significant injury or pathology and accurately predict morbidity and mortality. However, there are multiple challenges which must be overcome before HRV can become a routine monitoring and triage tool in trauma. The key issues for future investigations are

1. how to implement HRV in the triage of civilian and military trauma,
2. guidelines for the monitoring and assessment of trauma patients using HRV,
3. development of normal values and thresholds for treatment,
4. target values for resuscitation.

If HRV is to be a useful tool, real-time, simplified variability data must be made available to medics and physicians. Multiple wireless vital signs monitoring technologies are currently in development, several of which are able to provide continuous measurements of variability. Future trials integrating these devices into the triage and treatment of trauma patients will determine the clinical utility of HRV.

## Figures and Tables

**Table 1:** Definitions of time-domain metrics of heart rate variability.

Variable	Abbreviation	Description
Standard deviation of normal-normal RR intervals (NN)	SDNN	Measures the standard deviation of RRI for an entire measurement [3]
Standard deviation of average NN interval	SDANN	Measures the standard deviation of RRIs for short segments of ECG (usually 5 minutes) [3]
Root mean squared successive difference	RMSSD	Calculation of the square root of the mean squared differences in successive RRIs [3]
Proportion of successive NN intervals >50 ms	pNN50	Number of the number of interval differences of successive NN intervals >50 ms divided by total number of NN intervals [3]
Integer heart rate variability/heart rate volatility	HRVi	Calculation of the standard deviation of the integer heart rate for 5-minute increments [11, 15, 19–21]
Cardiac volatility-related dysfunction/cardiac uncoupling	CVRD	Percentage of time per 24-hour period that HRVi falls within a critically low range (0–0.5 or 0.3–0.6 bpm) [6, 12, 22]

**Table 2:** Definitions of frequency-domain metrics of heart rate variability.

Variable	Abbreviation	Description	Interpretation
Total power	TP	Total power of periodic oscillations in the ECG signal—represents total RR interval spectral power	Global measure of autonomic function [3]
High frequency power	HF	Power spectral density of high-frequency oscillations (0.15–0.4 Hz)	Function of respiratory rhythms under vagal (parasympathetic) regulation [18, 24, 31]
Low frequency power	LF	Power spectral density of low-frequency oscillations (0.04–0.15 Hz)	Baroreceptor reflex-related modulation of cardiac pacemaker activity via sympathetic and parasympathetic stimulation [4, 18, 31]
Very low frequency power	VLF	Power spectral density at very-low-frequency oscillations (<0.04 Hz)	Associated with vasomotor, thermal, and humoral regulation via sympathetic nerve activity [24, 31]
Low/high frequency ratio	LF/HF	Ratio of low-frequency power to high-frequency power	Represents ratio of sympathetic to parasympathetic nerve activity [31]

**Table 3:** Definitions of entropy metrics of heart rate variability.

Variable	Abbreviation	Description	Interpretation
Approximate entropy	ApEn	Measures the amount of irregularity in the RRI signal [40, 41]	Lower value reflects less complex signal
Sample entropy	SampEn		
Multiscale entropy	MSE	The sum of sample entropies [37]	

Similarity of distributions	SOD	The probability of similar RRI signal amplitude distributions as a function of time [33]	Higher SOD reflects more similarity and less complex regulation

Point correlation dimension	PD2i	Measures time-dependent changes in the degrees of freedom of a data set [33]	Lower value signifies loss of regulatory complexity

Fractal dimension by dispersion analysis	FDDA	Determines the fractal organization of the signal; measures self-similarity in the signal structure [41]	Lower value implies lower complexity of signal regulation
Fractal dimension by curve lengths	FDCL		

Detrended fluctuations analysis	DFA	Determines fractal-like correlation properties and uncovers short- and long-range correlations within the signal [42]	Distinguishes between fluctuations generated by complex systems and those arising from external stimuli

Signal stationarity	StatAv	Assesses whether the mean and SD of the signal change during time in each data set [8]	Lower value reflects a more stationary signal

Symbol dynamics entropy	SymDyn	Measures the probability of certain patterns within the RRI time series [33]	Lower value implies a more predictable signal with less complex regulation
Percentage of forbidden words	FW		
Symbol distribution entropy	DisnEn		

**Table 4:** Heart rate variability analysis techniques and their uses in trauma.

Technique	Description	Metrics	Evidence of changes in trauma	Population
Time domain	Estimation of variability using statistics and measures of central tendency [3]	HRVi	↓ predicts mortality, ICH, adrenal insufficiency [11, 15, 19–22, 36]	ICU
		Uncoupling	↑ reflects acidosis, coagulopathy, MOSF, AI, severe TBI, ↑ ICP, predicts mortality [6, 9, 12, 22]	ICU
		SDNN	↓ predicts TBI, mortality, acidosis, LSI [16, 23]	Prehospital ER ICU
		RMSSD	↓ reflects TBI, hemorrhage, mortality [23, 28, 47]	ER ICU

Frequency domain	Calculation of power (amplitude) of contributing frequencies to an underlying signal [18]	TP	↓ reflects ↑ICP, TBI, prolonged neurologic recovery, need for LSI, mortality, brain death [10, 28, 43]	ICU Outpatient
		LF	↓ reflects ↑ICP, TBI, hemorrhage, need for LSI, mortality, brain; ↑ reflects ↑CI, HR, MAP death [4, 10, 27, 28, 37, 43, 48, 49].	ER ICU
		HF	↓ reflects trauma, ↑ICP, need for LSI, hemorrhage, brain death, and mortality [10, 37, 43, 47, 50, 51]	Prehospital
		LF/HF	↑ reflects ↑ICP, ↓CPP; ↓ reflects brain death, mortality, hemorrhage, ↓GCS, poor neurologic outcome [5, 7, 10, 27, 48, 51]	Prehospital ICU

Entropy	Measurement of overall disorder, randomness, or irregularity of a physiologic signal [18]	MSE	↓ predicts mortality [13, 35, 36, 45]	ICU
		ApEn	↓ reflects trauma, burn, hemorrhage, brain death, and ↑ICP, MOSF, predicts mortality; ↑ reflects resuscitation [8, 14, 25, 29, 34, 37, 52]	Prehospital
		SampEn	↓ reflects trauma, mortality, burn, and hemorrhage; ↑ reflects resuscitation; predicts LSI [8, 25, 43]	Prehospital
		FDCL	↓ reflects hemorrhage; ↑ reflects resuscitation; ↓ predicts LSI [17]	Prehospital
		FDDA	↓ reflects need for LSI, hemorrhage, mortality [8, 25, 43]	Prehospital
		DFA	↓ reflects mortality; predicts LSI [8, 25, 43]	Prehospital
		SOD	↑ reflects need for LSI, mortality [8, 25, 43]	Prehospital
		StatAv	↓ reflects mortality [25]	Prehospital

HRVi: integer heart rate variability; ICH: intracranial hypertension; MOSF: multiple-organ system failure; AI: adrenal insufficiency; TBI: traumatic brain injury; SDNN: standard deviation of normal-normal RR Intervals (NN); RMSSD: root mean squared successive difference; LSI: life-saving intervention; ICP: intracranial pressure; TP: total power; LF: low-frequency oscillations power spectral density; HR: heart rate; CI: cardiac index; MAP: mean arterial pressure; HF: high-frequency oscillations power spectral density; LF/HF: low-to-high-power spectral density ratio; MSE: multiscale entropy; ApEn: approximate entropy; SampEn: sample entropy; SymDyn: symbol dynamics entropy; DisnEn: symbol distribution entropy; FDCL: fractional dimension by curve length; FDDA: fractional dimension by dispersion analysis; DFA: detrended fluctuations analysis; SOD: similarity of distributions; StatAv: signal stationarity; PS2i: point correlation dimension.
